# OLDER ADULTS WHO SCREEN POSITIVE FOR COGNITIVE IMPAIRMENT: PREDICTORS OF FOLLOW-UP

**DOI:** 10.1093/geroni/igad104.2754

**Published:** 2023-12-21

**Authors:** Matthew Cohen, Alyssa Lanzi, Anna Saylor, Aaron Boulton, Jennifer Rittereiser, Michelle Ritona, James Ellison

**Affiliations:** University of Delaware, Newark, Delaware, United States; University of Delaware, Newark, Delaware, United States; University of Delaware, Newark, Delaware, United States; University of Delaware, Newark, Delaware, United States; ChristianaCare, Wilmington, Delaware, United States; ChristianaCare, Wilmington, Delaware, United States; ChristianaCare, Wilmington, Delaware, United States

## Abstract

Interventions for cognitive decline are most likely to be effective when they are initiated as early as possible. Here, we present data from a community-based program for older adults that provides brain wellness education and cognitive screening. Individuals who screen positive are given the option to have results sent to their primary care provider (PCP). We have compared the characteristics of those who do with those who do not elect to send positive results to identify meaningful differences that might suggest program enhancements. 812 community-dwelling older adults (mean age = 73, SD = 10.7) were screened with the Mini-Cog and AD8 either in person (n = 534; 66%) or by Zoom (n = 278, 34 %). 367 (45.2%) participants screened positive. Of these, 279 (76%) elected to send their results while 82 (23%) declined. A logistic regression revealed that the decision to send results was not systematically related to age, sex, race, ethnicity, Mini-Cog score or AD8 score. It was, however, associated with the participant’s number of medical conditions (e.g., high blood pressure): OR = 1.293, p=.005. This indicates that people with greater awareness of their medical status, or those more actively engaged with a PCP are more comfortable communicating with their provider. Future research should investigate these and additional factors that might contribute to older adults’ decision to act on positive screening results. For example, participants may be more willing to share results if the screener emphasizes that (like blood pressure) some causes of cognitive impairment are modifiable.

